# RING-Type E3 Ubiquitin Ligases AtRDUF1 and AtRDUF2 Positively Regulate the Expression of *PR1* Gene and Pattern-Triggered Immunity

**DOI:** 10.3390/ijms232314525

**Published:** 2022-11-22

**Authors:** So Young Yi, Myungjin Lee, Suk-Yoon Kwon, Woo Taek Kim, Yong Pyo Lim, Si-Yong Kang

**Affiliations:** 1 Institute of Agricultural Science, Chungnam National University, Daejeon 34134, Republic of Korea; 2 Research Center of Crop Breeding for Omics and Artificial Intelligence, Kongju National University, Yesan 32439, Republic of Korea; 3 Plant Systems Engineering Research Center, Korea Research Institute of Bioscience and Biotechnology (KRIBB), Daejeon 34141, Republic of Korea; 4 Department of Systems Biology, Yonsei University, Seoul 03722, Republic of Korea; 5 Department of Horticulture, College of Agriculture and Life Science, Chungnam National University, Daejeon 34134, Republic of Korea; 6 Department of Horticulture, College of Industrial Sciences, Kongju National University, Yesan 32439, Republic of Korea

**Keywords:** pattern-triggered immunity, flagellin 22, RING-type E3 ubiquitin ligase, salicylic acid, NPR1, PR1, *Pseudomonas syringae* pv. *tomato*

## Abstract

The importance of E3 ubiquitin ligases from different families for plant immune signaling has been confirmed. Plant RING-type E3 ubiquitin ligases are members of the E3 ligase superfamily and have been shown to play positive or negative roles during the regulation of various steps of plant immunity. Here, we present Arabidopsis RING-type E3 ubiquitin ligases AtRDUF1 and AtRDUF2 which act as positive regulators of flg22- and SA-mediated defense signaling. Expression of *AtRDUF1* and *AtRDUF2* is induced by pathogen-associated molecular patterns (PAMPs) and pathogens. The *atrduf1* and *atrduf2* mutants displayed weakened responses when triggered by PAMPs. Immune responses, including oxidative burst, mitogen-activated protein kinase (MAPK) activity, and transcriptional activation of marker genes, were attenuated in the *atrduf1* and *atrduf2* mutants. The suppressed activation of PTI responses also resulted in enhanced susceptibility to bacterial pathogens. Interestingly, *atrduf1* and *atrduf2* mutants showed defects in SA-mediated or pathogen-mediated *PR1* expression; however, avirulent *Pseudomonas syringae* pv. *tomato* DC3000-induced cell death was unaffected. Our findings suggest that AtRDUF1 and AtRDUF2 are not just PTI-positive regulators but are also involved in SA-mediated *PR1* gene expression, which is important for resistance to *P. syringae*.

## 1. Introduction

Under natural conditions, plants are constantly challenged by abiotic and biotic stressors, and the degree of success with which the plants cope with these stressors reflects the effectiveness of their protective physical and chemical barriers. However, a complex signaling cascade of inducible defense responses is stimulated in the host on recognition of the pathogens that have overcome these constitutive defenses. The currently adopted zig-zag coevolutionary model between plant and pathogen suggests two branches of defense strategies [1,2]. First, the detection of conserved pathogen-/damage-/microbe-/herbivore-associated molecular patterns (PAMPs/DAMPs/MAMPs/HAMPs) by cognate plant cell-surface pattern recognition receptors (PRRs) triggers a major branch of innate immune (PTI) signaling in plants. The second branch, effector-triggered immunity (ETI) signaling, is activated by pathogen effector proteins, mostly via intracellular nucleotide-binding sites and leucine-rich repeat domain receptors (NLRs) [3,4,5,6]. Although the two classes of immune receptors are initiated by distinct activation mechanisms and involve different early signaling cascades, several studies have demonstrated that the activation of PRRs contributes to ETI. One of the early signaling molecules, reactive oxygen species (ROS), connects PTI to ETI [7,8]. Activated ETI amplifies the transcription and translation levels of key components of PTI. Furthermore, the components downstream of ETI play a significant role in PTI [7,8,9,10]. Lang et al. also reported that MPK3/6 activities could bridge PTI and ETI by positively controlling the SA sector of defense through the expression of NLR genes, and the ETI-regulating proteins non-race specific disease resistance1 (NDR1) and enhanced disease susceptibility 1 (EDS1) engage in this process [11].

Plant hormones are a group of naturally occurring organic substances that influence physiological processes, mainly growth, differentiation, and development, at low concentrations [12]. Obvious changes in hormone levels and different combinations of hormones functioning during different plant–microbe interactions function as efficient biological signals [13]. SA induces defense against biotrophic pathogens that feed and reproduce in live host cells [14]. Pathogen-induced accumulation of SA is an isochorismate synthase (ICS1)-dependent process [15]. A mutation in the *ICS1* gene (*SA induction–deficient (sid) mutant 2*) abolishes pathogen-induced SA accumulation and SAR [15,16]. Although ETI and PTI are induced by different types of pathogenic molecules, SA is the main hormone that mediates diverse immune responses in plants and is synthesized in response to a wide range of pathogens. The role of SA-mediated signaling in ETI was demonstrated in a study of SA-deficient mutants, showing that they support higher growth of the avirulent bacteria *Pst* DC3000 carrying *avrRpt2* [16]. In the case of PTI, resistance against *Pst* DC3000 is induced by bacterial PAMPs (flg22 and elf18) and is impaired in *sid2* [17]. RNAseq analysis of the response of Arabidopsis to SA revealed that SA treatment rapidly induces genes encoding PRRs, such as FLS2, EFR, CERK1, RLP23, and RLP30, and co-receptors, such as BKK1 and SOBIR1 [18,19]. SA is also involved in oxidative burst, a rapid and transient accumulation of ROS caused by FLS2–flg22 interaction [20]. Our previous report showed that, in the autoimmune mutant *cim6* in which SA signaling is constitutively active and SA levels are high [21], flg22-dependent generation of ROS is more pronounced than that in the wild-type (WT) control. In contrast, in *sid2* and *eds5* mutants [16,22] in which SA accumulation does not occur following exposure to biotic or abiotic stresses, the oxidative burst is much less pronounced than that in the WT control. nonexpressor of pathogenesis-related genes 1 (NPR1) is a master regulator of the defense-related genes induced by SA [23,24,25,26]. NPR1 interacts with the transcription factor TGACG SEQUENCE-SPECIFIC BINDING PROTEIN (TGA) TGA2/TGA5/TGA6, which functions redundantly in SA-induced *Pathogenesis-related* (*PR*) gene expression and disease resistance [27]. NPR1 functions as a transcriptional activator [28,29], and the binding of SA to NPR1 promotes its activity [18,30]. Kumar et al. provided a structural explanation for the direct role of SA in regulating NPR1-dependent gene expression through cryo-electron microscopy and crystal structure analysis of the NPR1 and TGA3 complex [31].

Protein ubiquitination is important for the regulation of plant immune signaling. The ubiquitin-related system ultimately ligates one or more ubiquitin molecules to specific target proteins through the sequential action of three enzymes, namely E1 (ubiquitin-activating), E2 (ubiquitin-conjugating), and E3 (ubiquitin ligase) [32]. E3 enzymes, which play essential roles in determining substrate specificity, are classified into the following four main subfamilies based on their subunit composition and mechanism of action: homologous to the E6-associated protein carboxyl terminus (HECT), really interesting new gene (RING), U-box, and cullin-RING ligases (CRLs) [32]. Among the 1300 E3 ubiquitin ligase genes in the Arabidopsis genome, more than 400 are predicted to encode RING-type enzymes [33]. *RING* gene expression in induced on treatment with an elicitor or inoculation with various pathogens [34,35,36]. Moreover, plants with altered RING-type E3 ubiquitin ligase gene expression levels (e.g., overexpression or silencing) exhibit modulated defense responses following pathogen infection [37]. These ubiquitin-related system components appear to influence all aspects of plant immunity from pathogen recognition to downstream signaling during PTI and ETI responses [38,39,40].

SA is involved in multiple defense processes including PTI and ETI. Over 40 E3 ubiquitin ligases are involved in plant immunity, but there are no reported instances of RING-type E3 ligases regulating both PTI- and SA-mediated signaling in Arabidopsis [39]. In this study, we investigated the functions of RING-type E3 ubiquitin ligases that are activated by pathogens and flg22 treatment. Knockout mutations in AtRDUF1 and AtRDUF2 resulted in suppressed flg22-triggered responses and enhanced bacterial growth relative to WT plants. Moreover, AtRDUFs are involved in the positive regulation of SA-mediated *PR1* gene expression. These results suggest that AtRDUF1 and AtRDUF2 participate in the positive regulation of PTI- and SA-mediated defense signaling in Arabidopsis.

## 2. Results

At5g59550 induced by flg22 was identified using in silico database analysis (http://www.genevestigator.com). At5g59550 is a RING-type E3 ubiquitin ligase that contains a DUF1117 motif in its C-terminal region. The RING-type E3 ligase gene with a DUF1117 motif is found in rice and Arabidopsis. Three such genes have been identified in Arabidopsis (At3g46620, At5g59550, and At2g39720) [33,41]. At3g46620 and At5g59550 were previously designated AtRDUF1 and AtRDUF2, respectively. Both proteins have been identified as ABA-, salt-, and drought-inducible RING finger domain-containing E3 ligases [42,43]. In the current study, we analyzed the expression patterns of three RING-type E3 ligase genes containing the DUF1117 motif after PAMP (flg22, elf18, and chitin) elicitation. FLS2 and RBOHD genes [44] were used as positive controls for PAMP treatment. Transcripts of AtRDUF1 and AtRDUF2 were accumulated within 30 min of PAMP treatment and were maintained at elevated levels until 1 h after flg22 treatment. The AtRDUF2 gene was rapidly expressed after treatment with all of the PAMPs used in this experiment, and it was maintained for 60 min; this expression pattern is similar to that of the FLS2 and RBOHD genes. Rapid but transient expression of AtRDUF1 was induced by elf18 and chitin (Figure S1). The PAMP-responsive gene expression pattern of At2g39720 differed from that of AtRDUF1 and AtRDUF2. It responded transiently to chitin treatment only (Figure S1). Based on these results, we selected the early flg22-response genes AtRDUF1 and AtRDUF2 from among three RING-type E3 ligases containing the DUF1117 motif to further study flg22-triggered immune signaling. 

### 2.1. PAMP Treatment Induces AtRDUF1 and AtRDUF2 Expression in Arabidopsis

**Figure 1:**
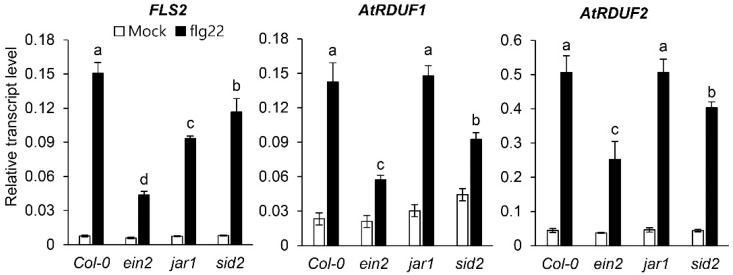
Flg22 induced expression of *AtRDUF1* and *AtRDUF2* in Arabidopsis mutants. Before performing quantitative reverse transcription polymerase chain reaction (qRT-PCR) analysis, 8-day-old seedlings were treated with 1 µM flg22 or H_2_O for 1 h. Transcript levels of FLS2, AtRDUF1, and AtRDUF2 were measured in wild-type (WT) (Col-0), *ein2*, *jar1*, and *sid2* seedlings, with transcript levels normalized to that of *ACT2*. Error bars represent the standard deviation of three replicates. Similar results were obtained in at least two independent experiments. Different letters indicate significant differences among plant genotypes (α = 0.05, one-way ANOVA and Tukey’s HSD test; JMP 15 software).

Plant defenses against pathogens are primarily regulated by three hormones: SA, jasmonic acid (JA), and ethylene (ET) [45]. In order to identify the plant defense hormones involved in early flg22-induced AtRDUF1 and AtRDUF2 expression, we analyzed the flg22 response in WT plants and known ET-, JA-, and SA-related mutant plants. Previous studies have indicated that EIN2, JAR1, and SID2 are essential for ethylene signal transduction [46], the production of biologically active jasmonoyl-L-isoleucine conjugates [47], and SA biosynthesis in response to pathogen attack [15], respectively. In previous studies, FLS2 has been used as a positive control for PAMP treatment [20,44]. As expected, the transcript levels of FLS2 after flg22 treatment were lower in mutant plants with abnormal hormone signaling than in WT plants (Col-0) (Figure 1). Similar to FLS2, the expression pattern of AtRDUF1 and AtRDUF2 in response to flg22 treatment was suppressed in mutant plants with defective ET or SA signaling, however, no alterations in the expression patterns were detected in jar1 mutant plants compared with WT plants (Figure 1). These data show that ET- and SA-dependent signaling (and not JA-mediated signaling) may be involved in flg22-induced AtRDUFs expression. 

### 2.2. Loss-of-Function Mutations of AtRDUFs Resulted in Suppressed Responses toward flg22

**Figure 2:**
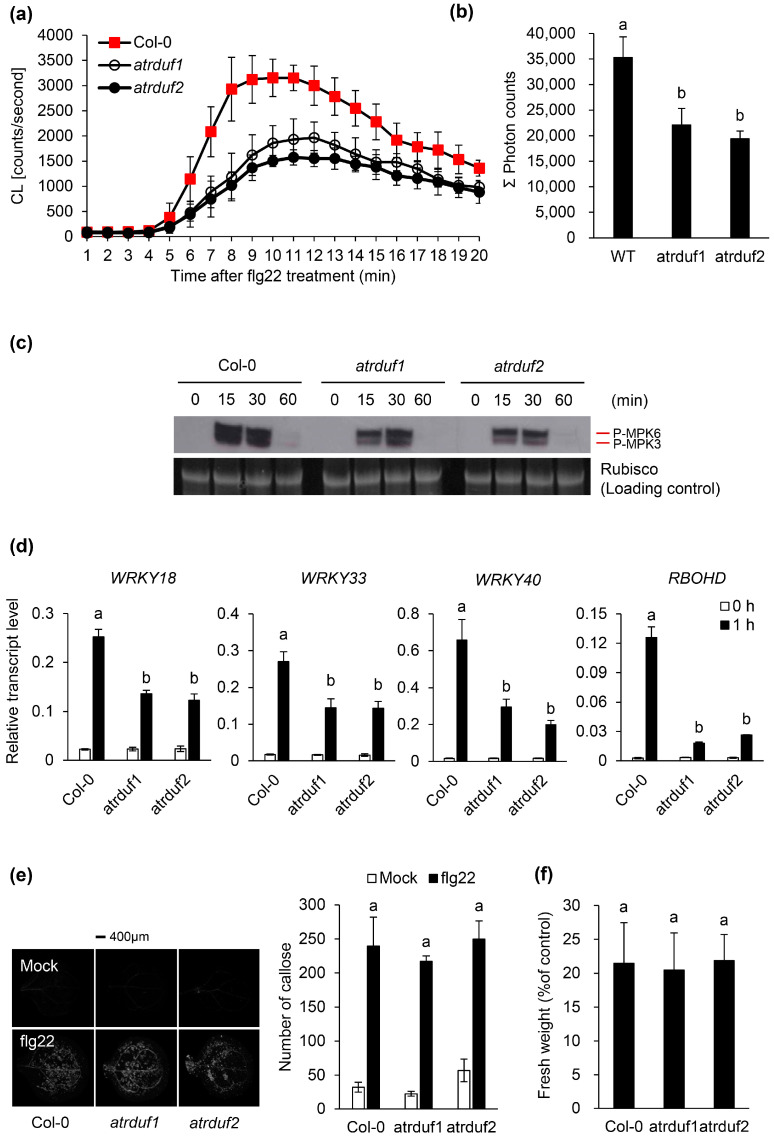
Functions of AtRDUF1 and AtRDUF2 during flg22-triggered immune signaling. (**a**) Flg22-induced reactive oxygen species (ROS) generation in liquid-grown intact seedlings of indicated Arabidopsis genotypes after treatment with 1 µM flg22. Error bars represent standard deviations of eight independent samples. Similar results were obtained in two independent experiments. (**b**) Flg22-induced ROS production in the indicated genotypes is represented as the integrated area under the ROS curve measured during a time course of 20 min and is referred to as Σ photon counts. Values are the mean ± SD (*n* = 8). (**c**) Dual phosphorylation of TEY motif in MPK3 and MPK6 in 8-day-old seedlings. Phosphorylated mitogen-activated protein kinases (MAPKs) corresponding to MPK3 and MPK6 are indicated. Activated MAPKs were detected by immunoblotting using antibody against Phospho-p44/42 MAPK (Erk1/2) (Cell Signaling Technology). The experiment was performed three times with similar results. Prior to transfer to polyvinylidene difluoride (PVDF) membrane, equal protein loading was checked by comparing the fluorescence intensity of Rubisco in stain-free gels. (**d**) Transcript levels of pathogen-associated molecular pattern (PAMP)-induced genes in Col-0, *atrduf1*, and *atrduf2* seedlings. Eight-day-old Arabidopsis seedlings were treated with 1 µM flg22 for 1 h, and transcript levels of *WRKY18*, *WRKY33*, *WRKY40*, and *RBOHD* were determined by quantitative reverse transcription polymerase chain reaction (qRT-PCR). Gene transcript levels were normalized to that of *ACT2*. Error bars represent the standard deviations of three replications. Similar results were obtained in at least two independent experiments. Different letters indicate significant differences among plant genotypes (α = 0.05, one-way ANOVA and Tukey’s HSD test; JMP software). (**e**) Flg22-induced callose deposition. Indicated phenotypes were either mock-treated or treated with 1 µM flg22 solution for 24 h. The leaves were then stained with aniline blue. Representative images were derived from 10 leaves of 10 independent plants. Similar results were obtained in three independent experiments. The number of callose depositions per cotyledon surface was automatically detected and analyzed using Photoshop CS6 software. This experiment was repeated three times with similar results. Values are mean ± standard error. (**f**) Flg22-induced seedling growth inhibition. Seedling growth of wild-type (WT) and *atrduf* mutants after flg22 treatment (1 µM). Four days post-germination, 10 seedlings of each genotype were transferred to 12-well plates containing half-strength Murashige and Skoog (MS) liquid media with or without 1 µM flg22. Seedlings were grown for a further 6 days and weighed. Similar results were obtained in four independent experiments. Different letters indicate significant differences among different genotypes in each treatment (α = 0.05, one-way ANOVA and Tukey’s HSD; JMP 15 software).

Several cellular responses such as ion fluxes across the plasma membrane [48], oxidative bursts [49], mitogen-activated protein kinase (MAPK) activation [50], and calcium-dependent protein kinase activation [51] were detected within seconds or minutes of the perception of flg22 by FLS2. These responses are followed by the transcriptional reprogramming of more than 1000 flg22-responsive genes [52]. Late flg22 responses such as SA accumulation, callose deposition at the plant cell wall, and seedling growth arrest [53,54] appear hours or days after the perception of the initial flg22 stimulus. As flg22 induces the accumulation of *AtRDUF1* and *AtRDUF2* transcripts (Figure 1), we tested whether the knockout of AtRDUFs affects other flg22 responses. We analyzed both the early flg22 responses (i.e., oxidative burst, MAPK activation, and PTI marker gene expression) and the late flg22 responses (i.e., callose deposition and seedling growth retardation) in WT and atrduf mutant plants (Figure 2). The early flg22 responses in the *atrduf1* and *atrduf2* mutants were weaker than those in the WT control (Figure 2a–d). The oxidative burst induced by flg22 was weaker in the *atrduf1* and *atrduf2* mutants than in the WT plants (Figure 2a,b), suggesting that these RING-type E3 ubiquitin ligases help regulate flg22-triggered oxidative burst. As expected, MPK3 and MPK6 were activated in the WT plants 15 min after flg22 treatment (Figure 2c). Interestingly, the *atrduf1* and *atrduf2* mutants showed suppressed flg22-induced MPK3 and MPK6 activation compared with WT controls (Figure 2c). We also analyzed the flg22-induced expression pattern of a set of PIGs [55]. We found that *WRKY18*, *WRKY33*, and *WRKY40* were downregulated in the *atrduf1* and *atrduf2* mutants and not in the WT (Figure 2d). Moreover, the genes encoding enzymes involved in the flg22-induced production of ROS, such as *RbohD* [56], were expressed at lower levels in the *atrduf1* or *atrduf2* mutants than in the WT plants (Figure 2d). These results imply that intact AtRDUF1 and AtRDUF2 are involved in the positive regulation of early flg22 responses. To further clarify whether AtRDUF1 and AtRDUF2 influence late flg22 responses, we treated *atrduf1* and *atrduf2* mutants with flg22. The resulting observed callose deposition and seedling growth arrest were similar between mutant and WT plants (Figure 2e,f). Hence, AtRDUF1 and AtRDUF2 appeared to regulate only early flg22-mediated responses. 

### 2.3. AtRDUF1 and AtRDUF2 Are Pathogen Inducible Genes and Positive Regulators of Pathogen- and SA-Mediated PR1 Gene Expression

**Figure 3:**
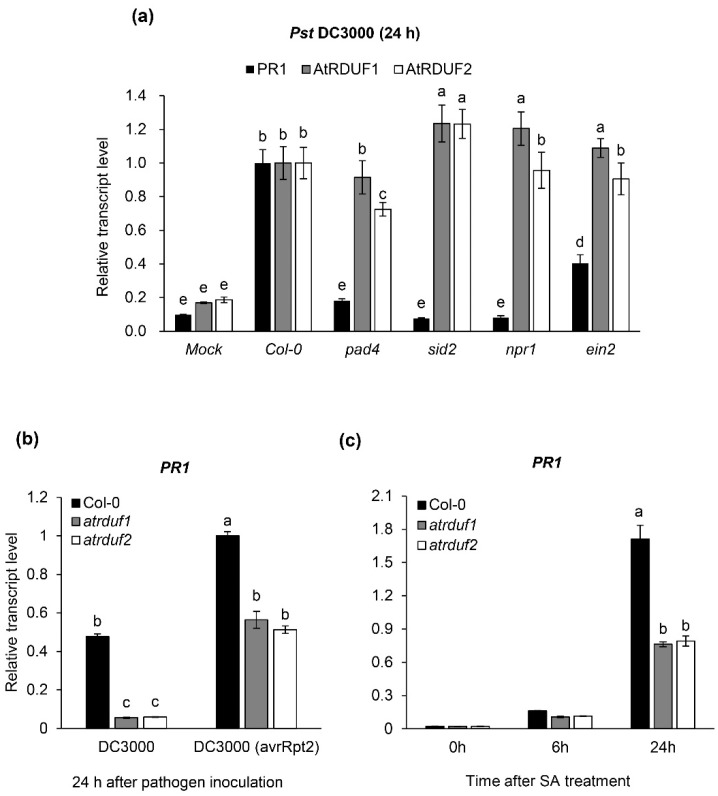
AtRDUF1 or AtRDUF2 are required for inducing *PR1* expression by pathogen or salicylic acid (SA). (**a**) Levels of *PR1*, *AtRDUF1*, and *AtRDUF2* transcripts were determined by quantitative reverse transcription polymerase chain reaction (qRT-PCR) in leaves of 5-week-old wild-type (WT) control, *pad4*, *sid2*, *npr1*, and *ein2* mutants with transcript level normalized against that of *ACT2*. Leaves of Arabidopsis were harvested 24 h post inoculation for extracting total RNA. Error bars represent standard deviations of three replications. Similar results were obtained in at least three independent experiments. Different letters indicate significant differences among plant genotypes after inoculation (α = 0.05, one-way ANOVA and Tukey’s HSD test; JMP software). (**b**) The transcript levels of *PR1* were determined using quantitative reverse transcription polymerase chain reaction (qRT-PCR) in leaves of 5-week-old wild-type (WT) control, *atrduf1*, and *atrduf2* mutants with transcript levels normalized against those of *ACT2*. Leaves of Arabidopsis were harvested 24 h post inoculation with *Pst* DC3000 or *Pst* DC3000 (*avrRpt2*) for extracting total RNA. Error bars represent the standard deviations of three replications. Different letters indicate significant differences among plant genotypes (α = 0.05, one-way ANOVA and Tukey’s HSD test; JMP software). (**c**) Levels of *PR1* transcript in wild-type (WT) and *atrduf* mutants after treatment with salicylic acid (SA) (1 mM). Leaves of Arabidopsis were harvested at 0, 6 h, and 24 h post treatment for extracting total RNA. Gene transcript levels were normalized to *ACT2* transcript levels. Error bars represent the standard deviations of three replications. Different letters indicate significant differences among plant genotypes (α = 0.05, one-way ANOVA and Tukey’s HSD test; JMP software).

In order to investigate *AtRDUF* transcript levels induced by pathogens, Arabidopsis leaves were inoculated with *Pst* DC3000. *PR1* gene expression is induced in response to various pathogens. Hence, we used the *PR1* gene as a molecular marker for infection [57,58] and found that it was distinctly upregulated 24 h after *Pst* DC3000 infection (Figure 3a). *Pst* DC3000 also increased the transcript levels of *AtRDUF1* and *AtRDUF2*, similar to that of *PR1* (Figure 3a). Flg22-induced expression of *AtRDUFs* was reduced in *sid2* or *ein2* mutants compared with that in WT plants. (Figure 1). Thus, to test the effect of phytohormones on pathogen-induced *AtRDUF* expression, we compared *Pst* DC3000-induced *AtRDUFs* expression in WT plants and plants with known mutated SA- or ET-mediated signaling. The *pad4*, *sid2*, and *npr1* mutants with impaired SA signaling [59] and *ein2* mutants with impaired ET signaling [46] were used for expression analysis. Interestingly, the pathogen-induced expression level of *AtRDUFs* in mutant plants with impaired SA- or ET-signaling was similar to or slightly higher than that of the WT. In contrast, the expression levels of *PR1* were significantly suppressed or unresponsive to infection in the plants with mutated SA- or ET-signaling when compared with the WT plants (Figure 3a). These results show that *AtRDUFs* are *Pst* DC3000-induced genes; however, unlike *PR1*, their expression can be regulated independent of SA- or ET-mediated signaling. *PR1* is a well-known defense marker that is strongly responsive to SA accumulation upon pathogen attack [57]. In order to investigate the effects of *AtRDUF* mutations on *PR1* transcript levels during compatible and incompatible interactions with pathogens, WT and *atrduf* mutant plants were inoculated with virulent (*Pst* DC3000) and avirulent (*Pst* DC3000 *avrRpt2*) *P. syringae*. Both *atrduf1* and *atrduf2* mutants showed *PR1* downregulation (Figure 3b), suggesting that AtRDUFs function upstream of *PR1* in *Pst* DC3000-induced defense signaling and positively regulates *PR1* expression. Furthermore, to confirm the involvement of AtRDUFs in SA-mediated *PR1* expression, we compared *PR1* transcript levels in SA-treated WT and *atrduf* mutants. As shown in Figure 3c, *PR1* was highly upregulated 24 h after SA treatment in WT plants. *PR1* expression was induced by SA treatment in *atrduf* mutants, but the expression level was 50% of that in the WT. These results suggest that *AtRDUF1* and *AtRDUF2* are pathogen-inducible genes that may function as positive regulators of pathogen- and SA-mediated *PR1* gene expression.

### 2.4. AtRDUF1 and AtRDUF2 Are Positive Regulators of Disease Resistance to Pst DC3000 but Are Not Involved in the Regulation of Hypersensitive Cell Death

**Figure 4:**
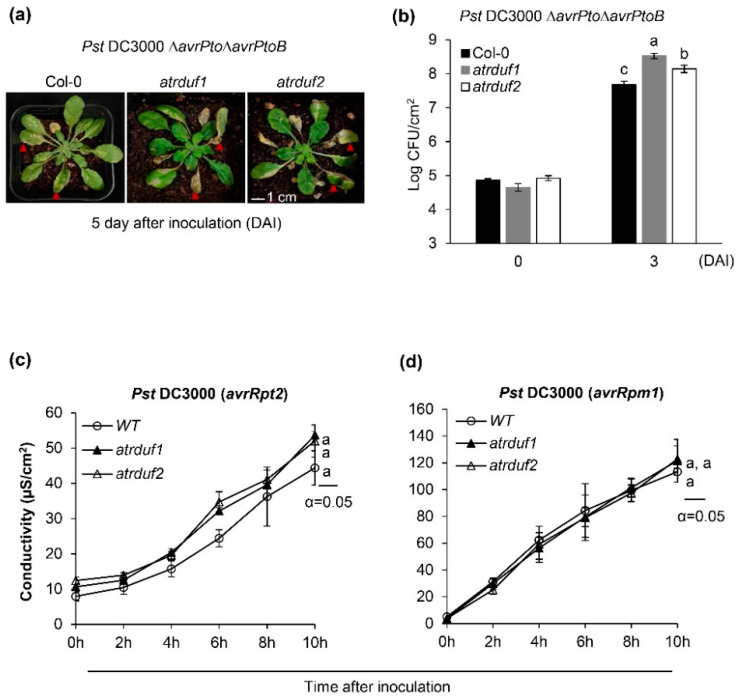
Functions of AtRDUF1 and AtRDUF2 during plant immune signaling. (**a**) Disease symptoms of wild-type (WT) Col-0 and *atrduf* mutants at 5 days after spray-inoculation with *Pst* DC3000 ∆*avrPto*∆*avrPtoB* (OD_600_ = 0.8). (**b**) Bacterial growth in Col-0 wild-type (WT) and *atrduf* mutants. Bacterial growth was determined at 0 and 3 days after inoculation (DAI). Error bars represent the standard deviation of five replications. Similar results were obtained in at least three independent experiments. Different letters indicate significant differences between plant genotypes at 3 days after inoculation (α = 0.05, one-way ANOVA and Tukey’s HSD test; JMP software). (**c**,**d**) Ion leakage assays of 5-week-old wild type (WT), *atrduf1*, and *atrduf2* mutant plants inoculated with avirulent strains of *Pst* DC3000 expressing *avrRpt2* (OD_600_ = 0.1) (**c**) or *avrRpm1* (OD_600_ = 0.1) (**d**). Error bars represent the standard deviation of three replicates. Similar results were obtained in at least two independent experiments. Different letters indicate significant differences between plant genotypes at 10 h post inoculation (α = 0.05, one-way ANOVA and Tukey’s HSD test; JMP 15 software).

In order to determine the effect of AtRDUF1 and AtRDUF2 on Arabidopsis immunity to *Pst* DC3000, we spray-inoculated WT and *atrduf* mutant plants with virulent strain *Pst* DC3000. No substantial differences in the disease symptoms and bacterial multiplication were detect between the *atrduf1* or *atrduf2* mutants and WT plants for 3–4 days after inoculation (Figure S2). Furthermore, we spray-inoculated WT and *atrduf* mutant plants using isogenic hypovirulent strains with deleted effectors AvrPto and AvrPtoB (*Pst* DC3000 *ΔavrPto/ΔavrPtoB*). Deletion of avrPto and avrPtoB in pathogenic *Pst* DC3000 has been reported to reduce its toxicity [60,61]. Unlike in the case of *Pst* DC3000, the bacterial count of *Pst* DC3000 *ΔavrPto/ΔavrPtoB* in *atrduf* mutant plants was almost 10-fold the count in WT plants (Figure 4a and Figure S2). Therefore, these results suggest that loss-of-function mutations in AtRDUFs result in enhanced susceptibility to *Pst DC3000 ΔavrPto/ΔavrPtoB.*

That the expression and functions of AtRDUFs are related to SA signaling components helps in elucidating their role in immune signaling. The activation of ETI leads to the upregulated expression of SA-activated genes, such as *PR1*, and is often associated with a hypersensitive response (HR) at the infection site [62]. To further elucidate the effect of *atrduf1* and *atrduf2* mutations on ETI, we assessed the HR induced by the *Pst* DC3000 strains *avrRpt2* and *avrRpm1*. The progression of cell death was quantified by measuring electrolyte leakage, which occurs upon cell death associated with an HR [63]. However, RPS2- and RPM1-dependent HR measurements indicated no statistically significant differences between the WT and *atrduf1* or *atrduf2* mutant plants in repeated experiments (Figure 4c,d). These results demonstrate that AtRDUF1 and AtRDUF2 are involved in SA-mediated *PR1* expression but have no effect on ETI signaling or cell death. To further characterize the function of AtRDUFs, we constructed transgenic plants overexpressing *AtRDUF2* under the control of the 35S CaMV promoter. We tried to confirm its function as a positive regulator of PTI or SA signaling through a gain-of-function study. Although *AtRDUF2-FLAG* transcripts were constitutively expressed in independently overexpressing transgenic lines (Figure S3a), the corresponding AtRDUF2-FLAG protein was undetectable in *35S:AtRDUF2-FLAG* plants using an anti-FLAG antibody (Figure S3b). this result was corroborated in previous studies, which found that AtRDUF1-sGFP and AtRDUF2-SGFP proteins were not expressed in transgenic *35S:AtRDUF-sGFP* overexpressing lines [42] and that proteins were degraded rapidly in transgenic Arabidopsis even though the RING E3 *HA-Rma* mRNA levels were high in the over-expressing lines [64].

## 3. Discussion

### 3.1. AtRDUF1 and AtRDUF2 Are PTI Signaling Components and Positively Regulate Immunity in Arabidopsis

The amplitude and duration of the flg22-triggered immune responses, unless tightly regulated, cannot ensure a suitable response. In this study, *atrduf1* and *atrduf2* loss-of-function mutants showed significantly defective ROS production, MAPK activation, and PIGs expression after flg22 elicitation (Figure 2). Thus, we tested whether weak early flg22 responses in the *atrduf1* and *atrduf2* mutants would lead to increased susceptibility to bacterial pathogens. We tested the virulent strain *Pst* DC3000 and the hypovirulent strain *Pst* DC3000 *ΔavrPto/ΔavrPtoB*, respectively [61]. Two effector proteins, AvrPto and AvrPtoB, share the same host target and manipulate multiple host factors [65]. AvrPto suppresses the kinase activity of FLS2, EFR, and AvrPtoB, which includes an E3 ubiquitin ligase domain and inhibits many PRRs, such as FLS2, FEN, CERK1, and Bti9 [66]. The results showed that the *atrduf1* and *atrduf2* mutants were significantly more susceptible than WT Col-0 to strains such as *Pst* DC3000 *ΔavrPto/ΔavrPtoB* with attenuated virulence, suggesting that attenuated FLS2-mediated defense signaling in *atrduf* mutants can enhance susceptibility to bacterial pathogens (Figure 4a and Figure S2). These results led us to speculate that AtRDUF1 and AtRDUF2 act as positive regulators of flg22-activated defense responses via the ubiquitination-mediated removal of the PTI signaling repressor, which is located upstream of MAPK cascades or ROS burst. Several reports have suggested that the enhanced activation of early flg22-triggered responses through the modulation of E3 ligases results in increased resistance against plant pathogens [67,68]. Both PUB12 and PUB13, which are highly homologous U-box E3 ligases, regulate FLS2 turnover. The flg22-triggered degradation of FLS2 is inhibited in *pub12/pub13* mutants, which exhibit enhanced flg22-induced oxidative burst and resistance against DC3000 [68]. Similar to the effect exerted by PUB12/PUB13, the PUB22/23/24 triplet negatively regulates PTI responses in Arabidopsis [67]. Regarding PUB12/13 and PUB22/23/24, the early flg22 response and pathogen resistance appeared to be positively correlated, and both groups of proteins were involved in the regulation of FLS2 turnover. 

### 3.2. Involvement of AtRDUFs in SA-Mediated Signaling during Early flg22-Triggered Immunity

In our previous study on an SA-deficient mutant *sid2*, we showed that basal SA levels contributed to early flg22-triggered responses. The Arabidopsis *sid2* mutant has a relatively low basal SA level and effectively suppressed flg22-triggered oxidative burst and *FLS2* expression compared with the WT [20]. However, the signaling components that link SA-mediated signaling and early flg22 responses in plant immunity regulation remain unclear. In the current study, expression analysis showed that *AtRDUF1* and *AtRDUF2* were activated in Arabidopsis by PAMPs (Figure 1 and Figure S1) and pathogens (Figure 3a). The *AtRDUF* transcript levels were significantly lower in the *sid2* mutant (as low as 60–80% of WT) 1 h after flg22 treatment. The suppression of flg22-induced expression of *AtRDUF1* and *AtRDUF2* in *sid2* was similar to that of *FLS2*, which was used as a control (Figure 1). An earlier study showed that SA accumulates significantly 3–6 h after flg22 treatment [69]. However, increased expression levels of PAMP-induced genes (PIGs), including *AtRDUF1*, *AtRDUF2*, and *FLS2*, were detected within 30 min of PAMP treatment (Figure S1). Thus, it is unlikely to be affected by *de novo* synthesis of SA as a result of the flg22 response. Hence, flg22 response-mediated induction of *AtRDUF1* and *AtRDUF2* expression is likely regulated by SA at a basal level. Flg22-induced *AtRDUF* transcript levels were also significantly lower in the *ein2* mutant than in Col-0 plants. Plants mutated in the key ethylene-signaling protein EIN2 show impaired FLS2-mediated responses, correlating with reduced *FLS2* transcription and protein accumulation [70]. AtRDUF1 and AtRDUF2 being downstream signaling components of FLS2 elicited by flg22, attenuated expression of *FLS2* could possibly reduce the expression of *AtRDUFs* in *ein2* (Figure 1). 

### 3.3. Involvement of AtRDUFs in SA-Mediated Signaling during Pathogen-Induced Immunity

Resistance to *Pst* DC3000 induced by flg22 pretreatment was compromised in *pad4* and *sid2* mutants, demonstrating that flg22-induced SA is important for flg22-triggered resistance [69]. As previously mentioned, *Pst* DC3000 induced *AtRDUF1*, *AtRDUF2*, and *PR1* expression 24 h after infection in WT plants (Figure 3a). To deduce if SA- or ET-signaling was related to pathogen-induced *AtRDUF* expression, we analyzed the differences in the expression patterns of *AtRDUFs* between the WT and mutants bearing mutations in the hormones (Figure 3a). Unlike *PR1*, which was used as a positive control, *AtRDUF1* and *AtRDUF2* showed pathogen-induced expression levels similar to those of WT in the SA-signaling mutant (*pad4*, *sid2*, and *npr1*) or the ET-signaling mutant *ein2* (Figure 3a). These results suggest that pathogen-induced *AtRDUF* expression is independent of SA and ET signaling mechanisms. We also tested whether AtRDUFs affect the control of SA-signaling. We analyzed the expression pattern of the SA-signaling marker gene *PR1* following treatment with virulent *Pst* DC3000, avirulent *Pst* DC3000 (*avrRpt2*), and SA in *atrduf1* or *atrduf2* mutants to determine the role of AtRDUFs in SA-mediated defense signaling (Figure 3b,c). Interestingly, pathogen- and SA-induced *PR1* in *atrduf1* and *atrduf2* mutants were significantly suppressed compared with WT. The results shown in Figure 3 indicate that AtRDUFs are components of SA-mediated signaling. Although pathogen-induced expression of *AtRDUF1* and *AtRDUF1* is independent of SA signaling (Figure 3a), these results imply that AtRDUF1 and AtRDUF2 are positive regulators of *PR1* expression during SA-mediated defense signaling (Figure 3b,c). When Arabidopsis is challenged by pathogens, an increase in SA biosynthesis generally occurs through transcriptional induction of the key synthetic enzyme isochorismate synthase 1 (ICS1) [15,71]. PAMPs activate transcription factors (TFs) such as WRKY28, TCP8/9, and NTL9, which promote the expression of enzymes ICS1 and SA biosynthesis [72,73]. NLRs signaling through the downstream components ENHANCED DISEASE SUSCEPTIBILITY1 (EDS1), PHYTOALEXIN DEFICIENT4 (PAD4), and ACTIVATED DISEASE RESISTANCE 1 (ADR1) upregulates ICS1 [74,75]. More recently, it has also been discovered that the EDS1–PAD4–ADR1 node is a convergence point for defense signaling cascades activated by both PTI and ETI in conferring pathogen immunity [9]. *AtRDUFs* are early flg22 response genes affected by basal SA (Figure 1 and Figure S1) and have a *Pst* DC3000-induced expression pattern similar to WT in *pad4* or *sid2* mutant plants (Figure 3a). However, in this study, we did not analyze whether *atrduf1* or *atrduf2* mutants have impaired pathogen-induced SA accumulation, so it is unknown whether the function of AtRDUFs in SA-mediated signaling is upstream of ICS1.

**Figure 5:**
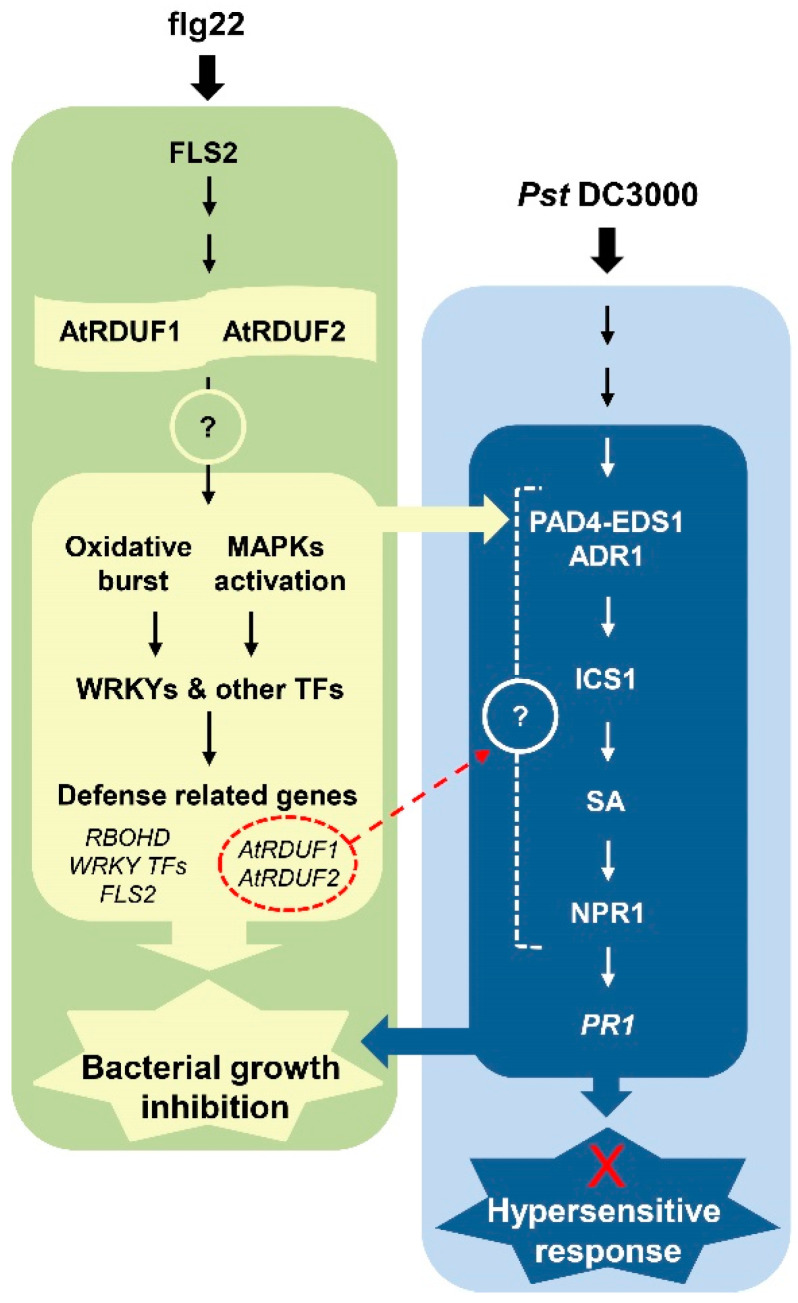
AtRDUF1 and AtRDUF2 are positive regulators of pathogen-associated molecular pattern-triggered immunity (PTI). Recognition of flg22 or pathogens activates AtRDUF1 and AtRDUF2 to amplify flg22-triggered immune responses by removing putative target proteins (question mark in the yellow green circle). At a later time point, activated early flg22-triggered immune responses support ICS1 mediated production of salicylic acid (SA), which leads to the activation of plant immune signaling [54]. During activated SA signaling, AtRDUF1 and AtRDUF2 activate *PR1* expression by interacting with a putative target protein (question marked in blue circles). Knockout mutants of AtrDUF1 or AtRDUF2 showed enhanced susceptibility to *Pst* DC3000. AtRDUF1 and AtRDUF2 are involved in SA-mediated *PR1* expression but have no effect on cell death. The pathogen-induced expression of AtRDUF1 and AtRDUF2 does not involve the known defense signaling components ICS1, PAD4, and NPR1. TFs, transcription factors.

The signal transduction pathway downstream of SA has been characterized by analysis of the *npr1* mutant [23,76]. NPR1 contains an N-terminal BTB/POZ domain, central ankyrin-repeat domain, and C-terminal transactivation domain [29,76]. Arabidopsis *npr1* mutants fail to respond to various SAR-inducing treatments, display little expression of pathogenesis-related (PR) genes, and exhibit increased susceptibility to infections because the ankyrin consensus sequence required for the transcriptional cofactor activity of NPR1 is impaired [76]. A recent study provided a structural explanation for the direct role of SA in regulating NPR1-dependent gene expression. After stimulation, SA-induced folding and docking of the SA-binding domain onto ankyrin repeats is required for the transcriptional cofactor activity of NPR1 [31]. In the current study, exogenous SA induced *PR1* expression in WT plants; therefore, it can be estimated that it was sufficient to activate the transcriptional cofactor NPR1 (Figure 3c). At this time, *atrduf1* and *atrduf2* mutants had lower SA-induced *PR1* expression levels than the WT (Figure 3c). Thus, the target proteins of AtRDUFs in SA-mediated signaling may be repressors of *PR1* expression. When intact E3 ubiquitin ligase AtRDUFs participate in the removal of putative targets, the *PR1* transcript level can be elevated. If that is the case, as AtRDUFs could positively regulate early flg22-triggered-immune response, it remains unclear whether AtRDUFs have multiple target proteins or whether they regulate the same target protein (related to plant immune signals) at different times. Zheng et al. demonstrated that SA biosynthesis is regulated by multiple TFs spatially and temporally [72]. As AtRDUFs engage in both basal SA-regulated response and activated SA signaling-mediated responses, we may hypothesize that they have multiple target proteins (Figure 5). 

Figure 5 shows a model of the position of AtRDUF1 and AtRDUF2 in the defense signaling network. Recognition of flg22 by FLS2 activates a MAP kinase cascade, which in turn activates changes in gene expression. Flg22 recognition also triggers elevation of reactive oxygen species (ROS). Activated AtRDUF1 and AtRDUF2 positively regulate early flg22-triggered immune responses (MAPK activation, ROS production, and defense-related gene induction). Flg22 signaling also activates SA production, and the activation of SA signaling by flg22 is important for flg22-induced resistance [20,69]. SA signaling is also activated in response to the recognition of effectors by NLRs (ETI). One might ask at what point AtRDUF1 and AtRDUF2 function affects SA signaling. The signals from AtRDUF1 and AtRDUF2 must act upstream of *PR1* expression, as SA-mediated *PR1* expression levels are reduced in *atrduf1* and *atrduf2* mutants. However, the positions of AtRDUF1 and AtRDUF2 in canonical SA-mediated signaling remain to be elucidated (Figure 5). Among the RING-type E3 ubiquitin ligases, very few are implicated in both plant immunity and SA signaling, and they are involved in ETI signaling rather than in PTI. RING1, a RING-finger E3 ligase protein, is required for cell death and SA-dependent defense response [77,78,79,80]. BAH1/NLA plays a crucial role in ubiquitination-mediated regulation of immune responses, including pathogen-induced SA accumulation and control of cell death [81]. Since our data showed that the RING-type E3 ligases, AtRDUF1, and AtRDUF2 are positive regulators of PTI and SA signaling, we expected that they might play a role in HR regulation. Additionally, a recent report demonstrated that the hypersensitive response that depends on intracellular receptors is strongly enhanced by the activation of surface receptors [7]. However, HR induced by *atrduf* mutations and avirulent pathogens (*Pst* DC3000 *avrRpt2* or *Pst* DC3000 *avrRpm1*) led to interactions that were similar to that in the WT (Figure 4b,c and Figure 5). These results suggest that AtRDUFs acts as positive regulators of SA-mediated PR1 expression but may not be involved in HR cell death in ETI signaling. Further research is required to identify the target proteins of AtRDUF1 and AtRDUF2 during PTI in Arabidopsis (Figure 5). Suppression of flg22-triggered immune response or pathogen-induced *PR1* expression could be a reason for enhanced bacterial growth in the *atrduf1* and *atrduf2* mutants. Future studies are needed to explore this, and it will be interesting to determine the nature of the interactions of AtRDUF1 and AtRDUF2 with various plant immune signaling components in Arabidopsis.

## 4. Materials and Methods

### 4.1. Plant Material and Growth Conditions

Arabidopsis plants were grown on soil in a growth chamber at 23 °C with a 16 h light/8 h dark photoperiod for long-day conditions and an 8 h light/16 h dark photoperiod for short-day conditions. To grow Arabidopsis seedlings on Murashige and Skoog (MS) medium, the seeds were surface sterilized using a gas sterilization method and germinated on sterile half-strength MS medium (pH 5.7) supplemented with 1% (*w*/*v*) sucrose and 0.6% (*w*/*v*) agar. Plated seedlings were grown in a growth chamber at 23 °C with a 16 h light/8 h dark photoperiod. All Arabidopsis mutants used in this study were from the Columbia (Col-0) background. The *atrduf1*, *atrduf2*, *ein2*, *pad4*, *sid2*, and *npr1* mutants were generated previously [20,42].

### 4.2. Pathogen Inoculation and Chemical Treatments

In order to generate bacterial growth curves, 6-week-old Arabidopsis plants were spray-inoculated with a bacterial solution at an OD_600_ of 0.8 as described by Zipfel et al. (2004). Briefly, DC3000 and its mutant derivatives were cultured at 30 °C in Luria-Bertani (LB) medium supplemented with appropriate antibiotics until the OD_600_ was attained. Bacteria were collected by centrifugation and resuspended in a solution containing 10 mM MgCl_2_ and 0.02% (*v*/*v*) Silwet L-77 (OSi Specialties, Tarrytown, NY, USA) to achieve a final concentration of 1 × 10^8^ CFU mL^−1^ (OD_600_ = 0.8). Arabidopsis plants were sprayed with the bacterial suspension and kept under high humidity until the development of disease symptoms. Exogenous chemicals were applied at the following concentrations: 1 µM flg22, elf18 (Peptron, http://www.peptron.com), chitin (*hexa*-N-acetyl-chitohexaose, Megazyme, www.megazyme.com), and 1 mM SA (Sigma-Aldrich, St Louis, MO, USA).

### 4.3. MAPK Phosphorylation Assay and Protein Detection

MAPK activity was determined using crude protein extracts from 8-day-old seedlings treated with 1 µM flg22 for 15–60 min as previously described [82]. Crude extracts were separated using 12% SDS-PAGE, and proteins were transferred to a polyvinylidene difluoride (PVDF) membrane (Bio-Rad, www.bio-rad.com) using semi-dry electroblotting (Mini-Protean II system; Bio-Rad). Activated MAPKs were detected following 1 h incubation with Phospho-p44/42 MAPK (Erk1/2) rabbit monoclonal antibody (mAb) (1:2000; Cell Signaling Technology, www.cellsignal.com), followed by subsequent 1 h incubation with anti-rabbit-HRP secondary antibody (Bio-Rad). The signals were visualized using an enhanced chemiluminescence system (Clarity Western ECL; Bio-Rad). Protein detection and immunoblot analyses were performed according to the manufacturer’s instructions using mouse anti-FLAG as the primary antibody (M2, diluted 1/500; F3165, Sigma–Aldrich, St Louis, MO, USA). The secondary antibody was labeled with anti-Mouse–HRP secondary antibody (Bio-Rad) and diluted to 1/1000.

### 4.4. Seedling Growth Inhibition

Flg22-induced seedling growth inhibition assays [53] were performed as described previously [83]. Approximately 10 Arabidopsis seedlings per treatment were grown on agar-solidified half-strength MS medium containing vitamins (Duchefa, www.duchefa-biochemie.com) and 1% (*w*/*v*) sucrose for 4 days and transferred to 12-well plates containing liquid half-strength MS medium supplemented with vitamins and 1% (*w*/*v*) sucrose with or without 1 µM flg22 peptide. Seedling fresh weight was recorded 6 days later.

### 4.5. Ion Leakage Assay

Ion leakage assays were conducted after syringe infiltration of plants with virulent DC3000 strains (*avrRpt2* or *avrRpm1*), as previously described [84] with minor modifications. Immediately after infiltration, 20 leaf discs were collected from five plants with a cork borer (r = 4 mm) and floated adaxially in 50 mL distilled water. After 15 min, the discs were transferred to 25 mL of fresh water to measure conductance.

### 4.6. Measurement of ROS Generation

The ROS content of 8-day-old seedlings was measured. The flg22-triggered oxidative burst was examined as previously described [20] with minor modifications. Briefly, the seedlings were incubated in a 96-well microplate containing liquid MS medium supplemented with 0.1% (*w*/*v*) sucrose. A multi-label reader, EnVision 2101 (Perkin Elmer, Waltham, MA, USA), was used to verify L-012-derived chemiluminescence (counts per second) at an emission wavelength of 590 nm.

### 4.7. Aniline Blue Staining, Microscopy Analysis, and Callose Quantification

Seedlings were collected, stored in 95% ethanol, and stained with aniline blue as previously described [53] with minor modifications. Briefly, seedlings were incubated for at least 24 h in 95–100% ethanol until all tissues were transparent. They were then washed with 0.07 M phosphate buffer (pH = 9) and incubated for 1–2 h in 0.07 M phosphate buffer containing 0.01% (*w*/*v*) aniline blue (Sigma) prior to the microscopic analysis. A minimum of eight cotyledons per condition per experiment were visualized under ultraviolet light using an epifluorescence microscope (TE 2000 Nikon, Tokyo, Japan). Callose was quantified from digital photographs by the number of white pixels (callose intensity) or the number of depositions relative to the total number of pixels covering the plant material using Photoshop CS6 software [85].

### 4.8. Quantitative RT-PCR Analysis

Total RNA was isolated from the collected seedlings using a Spectrum™ Plant Total RNA Kit (Sigma). Approximately 1 µg of DNA-free RNA was used as the template for first-strand cDNA synthesis using M-MLV reverse transcriptase (TOYOBO, www.toyobo-global.com). A qRT-PCR assay was conducted using the CFX96 qPCR system (Bio-Rad) and a 20 µL reaction solution that included TB Green™ Premix Ex Taq II (TaKaRa, www.takarabio.com) and primers (0.1 µM). The qRT-PCR program was as follows: 95 °C for 30 s; and 40 cycles of 95 °C for 5 s and 60 °C for 20 s. Subsequently, the dissociation curve was generated. All reactions were performed in triplicate. Details regarding the qRT-PCR primers and accession numbers of the analyzed genes are provided in Table S1.

### 4.9. Generation of Transgenic Arabidopsis Lines

The full coding sequence of each gene was amplified, and PCR products were cloned into the pGEM-T-easy vector (Promega, Madison, WI, USA) and transferred to the pCAMBIA1390 vector via the *Sal*I and *EcoR*I sites. To generate *AtRDUF2*-overexpression lines, the *AtRDUF1* protein-coding sequence was cloned into the pCAMBIA-Flag vector via the *Sal*I and *Eco*RI sites. Arabidopsis (Col-0) plants were transformed by the floral-dip method [86] using *Agrobacterium tumefaciens* strain GV3101. More than 20 independent T1 lines were generated for each construct. Three T2 lines were selected for T3 propagation based on gene expression levels. The seed batch showing 100% hygromycin resistance was confirmed as the homozygous T3 generation, and T3 homozygous seeds were used for the analyses.

## Data Availability

The data supporting the findings of this study are available from the corresponding author upon reasonable request.

## Supplementary Materials

The following supporting information can be downloaded at: https://www.mdpi.com/article/10.3390/ijms232314525/s1. References [87,88,89,90] are cited in the supplementary materials.
