# Supplementary material for: RING-Type E3 Ubiquitin Ligases AtRDUF1 and AtRDUF2 Positively Regulate the Expression of PR1 Gene and Pattern-Triggered Immunity
Source: Int J Mol Sci. 2022 Nov 22;23(23):14525. doi: 10.3390/ijms232314525 (PMC9739713; doi:10.3390/ijms232314525)
Supplement: Supplementary file 1 [file ijms-23-14525-s001.zip › ijms-1927206-supplementary/supplementary figures.pdf]

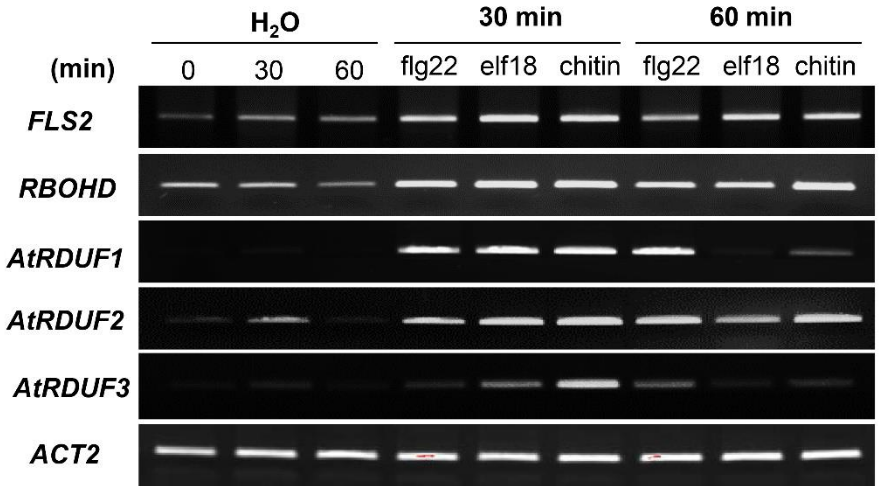

Figure S1. Pathogen-associated molecular patterns (PAMPs) induced gene induction.

Eight-day-old Arabidopsis Col-0 seedlings were either mock-treated (H_2O) or treated with 1 μM pathogen-associated molecular patterns (PAMPs) (flg22, elf18, and chitin) for 30 and 60 min, respectively. Gene expression was analyzed using semi-quantitative reverse transcription polymerase chain reaction (RT-PCR). *ACT2* was used as a standard control to equalize the amount of cDNA used in each reaction. The experiments were conducted twice, and similar results were obtained. *FLS2*, AT5G46330; *RBOHD*, At5g47910; *AtRDUF1*, At3g46620; *AtRDUF2*, At5g59550; *AtRDUF3*, At2g39720; *ACT2*, At3g18780;

(a)

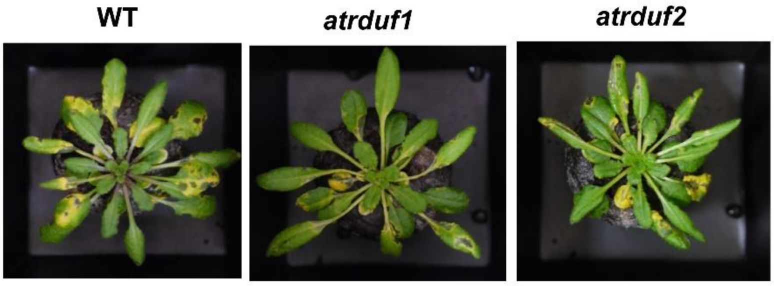

(b)

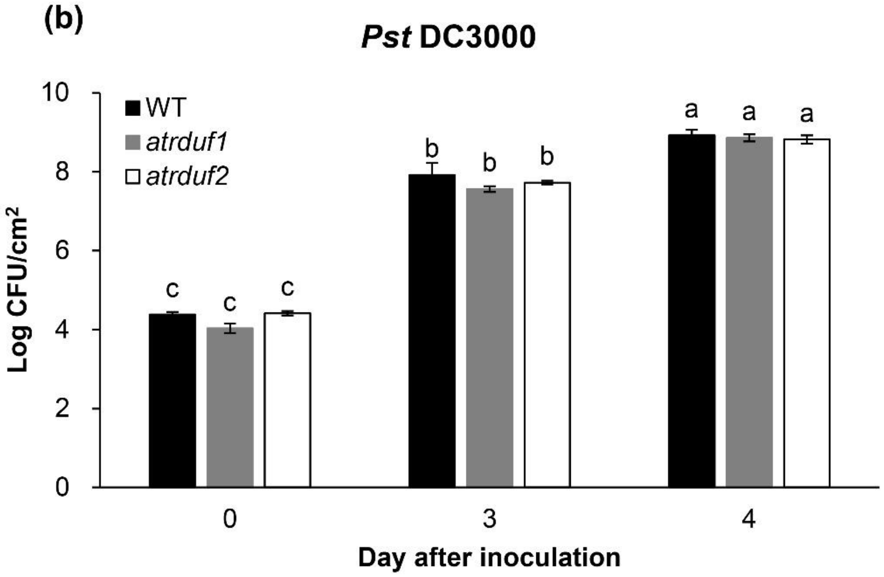

Figure S2. Results of bacterial infection assays with *Pst* DC3000.

(a) Disease symptoms of wild-type (WT) Col-0 and *atrduf* mutants on day 5 after spray-inoculation with *Pseudomonas syringae* pv. *tomato* DC3000 (OD_{600} = 0.8).

(b) Bacterial growth in Col-0 (WT) and *atrduf* mutants. Bacterial growth was determined at 0, 3, and 4 days post inoculation. Error bars represent standard deviation of five replications. Similar results were obtained in at least three independent experiments. Different letters indicate significant differences between plant genotypes on day 3 and 4 after inoculation (α=0.05, one-way ANOVA and Tukey's HSD test; JMP software).

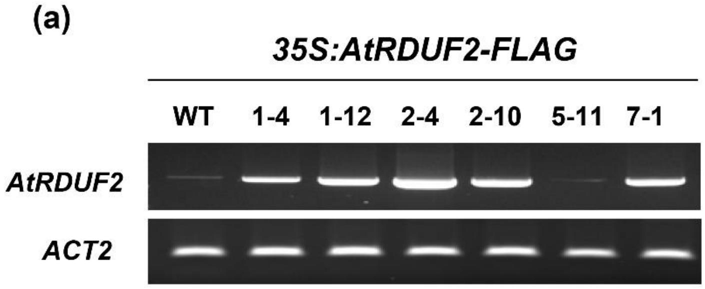

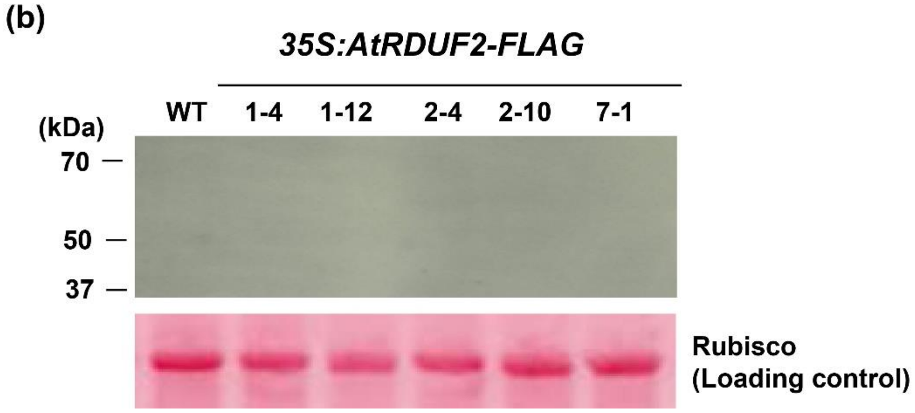

Figure S3. Characterization of transgenic plants overexpressing *35S:AtRDUF2-FLAG*.

(a) Gene expression was analyzed by semi-quantitative reverse transcription polymerase chain reaction (RT-PCR). *ACT2* was used as standard control to equalize amount of cDNA in each reaction. Transcript levels of *AtRDUF2-FLAG* in wild-type (WT) and T3 *35S:AtRDUF2-FLAG* (independent lines #1-4, #1-12, #2-4, #2-10, #5-11, and #7-1) transgenic plants were determined.

(b) Immuno-blot analysis of WT and *35S:AtRDUF2-FLAG* transgenic plants. Expression levels of AtRDUF2-FLAG protein were estimated with an anti-FLAG M2 antibody (Sigma). Equal loading was confirmed by Ponceau S staining of Rubisco (lower panels).
