# Supplementary material for: RING-Type E3 Ubiquitin Ligases AtRDUF1 and AtRDUF2 Positively Regulate the Expression of PR1 Gene and Pattern-Triggered Immunity
Source: Int J Mol Sci. 2022 Nov 22;23(23):14525. doi: 10.3390/ijms232314525 (PMC9739713; doi:10.3390/ijms232314525)
Supplement: Supplementary file 1 [file ijms-23-14525-s001.zip › ijms-1927206-supplementary/Table S1.pdf]

Table S1. List of primers for qRT-PCR

Gene	Locus	primer name	5'-primer-3'	Reference
AtRDUF1	At3g46620	Q-RDUF1-F	TTGATTTGGATAGTGATGGTGAA	
		Q-RDUF1-R	TTGATGTAACAAGAGAGGTACCAAA	
AtRDUF2	At5g59550	Q-RDUF2-F	GGTTAGGTCACACGGTACACTCG	
		Q-RDUF2-R	TTGACCTATCCAAAACCCTAGATTC	
AtRDUF3	At2g39720	Q-RDUF3-F	ACGAAATTGGCTAGCGTGAT	
		Q-RDUF3-R	TCGCATAGATTCAAATTTTAGGAA	
ACT2	At3g18780	Q-ACT2-F	AGTGTCTGGATCGGTGGTTC	(Truman *et al.*, 2013)
		Q-ACT2-R	CCCCAGCTTTTTAAGCCTTT	
FLS2	At5g46330	Q-FLS2-F	ACTCTCCTCCAGGGGCTAAGGAT	(Boutrot *et al.*, 2010)
		Q-FLS2-R	AGCTAACAGCTCTCCAGGGATGG	
WRKY18	At4g31800	Q-WRKY18-F	CATACGAAGGGACGCATAAC	(Birkenbihl *et al.*, 2017)
		Q-WRKY18-R	CCTTTCGTTTTTCTCCAACG	
WRKY33	At2g38470	Q-WRKY33-F	CTCAAGCACCATATACACTTCA	(Birkenbihl et al., 2017)
		Q-WRKY33-R	CCTTTGCTCTAGAGAATCCACC	
WRKY40	At1g80840	Q-WRKY40-F	CTTGACTGTGCCGGTGACTA	(Birkenbihl et al., 2017)
		Q-WRKY40-R	GAAGAAGCCATTTGCTCCAC	
PR1	At2g14610	Q-PR1-F	TTCTTCCCTCGAAAGCTCAA	(Lai *et al.*, 2008)
		Q-PR1-R	AAGGCCCACCAGAGTGTATG	
RBOHD	At5g47910	Q-RBOHD-F	CTGGACACGTAAGCTCAGGA	(Plancot *et al.*, 2013)
		Q-RBOHD-R	GCCGAGACCTACGAGGAGTA	

Birkenbihl, R. P., Kracher, B., Roccaro, M. and Somssich, I. E. (2017) Induced genome-wide binding of three Arabidopsis WRKY transcription factors during early MAMP-triggered immunity (vol 29, pg 20, 2017). *Plant Cell,* **29,** 1175-1175.

Boutrot, F., Segonzac, C., Chang, K. N., Qiao, H., Ecker, J. R., Zipfel, C*., et al.* (2010) Direct transcriptional control of the Arabidopsis immune receptor FLS2 by the ethylene-dependent transcription factors EIN3 and EIL1. *Proc Natl Acad Sci U S A,* **107,** 14502-14507.

Lai, Z. B., Vinod, K., Zheng, Z. Y., Fan, B. F. and Chen, Z. X. (2008) Roles of Arabidopsis WRKY3 and WRKY4 transcription factors in plant responses to pathogens. *BMC Plant Biol.,* **8**.

Plancot, B., Santaella, C., Jaber, R., Kiefer-Meyer, M. C., Follet-Gueye, M. L., Leprince, J.*, et al.* (2013) Deciphering the Responses of Root Border-Like Cells of Arabidopsis and Flax to Pathogen-Derived Elicitors. *Plant physiology,* **163,** 1584-1597.

Truman, W., Sreekanta, S., Lu, Y., Bethke, G., Tsuda, K., Katagiri, F.*, et al.* (2013) The CALMODULIN-BINDING PROTEIN60 Family Includes Both Negative and Positive Regulators of Plant Immunity. *Plant physiology,* **163,** 1741-1751.
